# Supplementary material for: Circulating Proteins as Diagnostic Markers in Gastric Cancer
Source: Int J Mol Sci. 2023 Nov 29;24(23):16931. doi: 10.3390/ijms242316931 (PMC10706891; doi:10.3390/ijms242316931)
Supplement: Supplementary file 1 [file ijms-24-16931-s001.zip › ijms-2720287-supplementary.doc]

**Table S1.** List of circulating protein markers for GC diagnosis reported over the past 10 years.

| **Protein^(a)^** | **Biological fluid** | **Ref.** |
| --- | --- | --- |
| AAT | gastric juice | [38] |
| ADAM12 | urine | [94,95] |
| ADAM8 | serum | [81] |
| AFP | serum | [58,74] |
| AHSG | serum | [72] |
| ANGPTL2 | serum | [86] |
| ANXA11 | urine | [93] |
| ANXA2 | serum | [85] |
| APOA-I | serum | [72] |
| ApoC1 | plasma | [49] |
| BIRC5 | serum | [68] |
| C4-A | plasma | [49] |
| CA19.9 | serum | [58,62,71,74] |
| CA72-4 | serum | [70,71] |
| CA125 | serum | [58,74] |
| CA153 | serum | [58] |
| CA242 | serum | [58] |
| CA72-4 | serum | [60] |
| Cat S | serum | [79] |
| CD59 | serum | [69] |
| CDC42 | urine | [93] |
| CEA | serum | [56,58,62,70,71,74] |
| CLU-1 | serum | [78] |
| COF1 | serum | [69] |
| COPS2 | serum | [87] |
| CRP | serum | [52] |
| CSTB | saliva | [91] |
| CTSF | serum | [87] |
| CXCL8 | serum | [62] |
| CXCR2 | serum | [62] |
| CystD | gastric juice | [89] |
| DcR3 | serum | [75] |
| DEK | plasma | [48] |
| DKK1 | serum | [60] |
| DMBT1 | saliva | [91] |
| EL | urine | [96] |
| Ela3A | gastric juice | [89] |
| ENOSF1 | serum | [87] |
| EphA1 | serum | [67] |
| EREG | serum | [67] |
| FGA | serum | [72,82] |
| FGF‐12 | serum | [67] |
| FR‐b | serum | [67] |
| G-17 | serum | [53] |
| Galectin‐8 | serum | [67] |
| GastL | gastric juice | [89] |
| Gastricsin | gastric juice | [89] |
| GHR | serum | [67] |
| GIF | gastric juice | [38] |
| GKN1 | serum | [66] |
| GPX3 | serum | [52] |
| GSN | plasma | [49] |
| HLA-G | plasma | [43,50] |
| IFNGR1 | serum | [67] |
| IGF-1 | serum | [54] |
| IL-6 | serum | [55,56,70] |
| IL-8 | serum | [51] |
| IL-16 | serum | [23] |
| IL-18 | serum | [84] |
| IL-2R | serum | [76] |
| Integrin α5 | serum | [67] |
| ITGB6 | serum | [52] |
| ITIH4 | serum | [63,69,83] |
| Leptin | serum | [73] |
| MMP-9/NGAL | urine | [95] |
| NAPA | urine | [93] |
| Notch‐3 | serum | [67] |
| NT5E | serum | [87] |
| PD-1 | plasma | [45,61] |
| PD-L1 | serum | [45,61] |
| PepA | gastric juice | [89] |
| PGC | ascitic fluid | [88] |
| PGI | serum | [53,55,81] |
| PGII | serum | [53,55,81] |
| PGR | serum | [53,55] |
| POSTN | ascitic fluid | [88] |
| S100A8 | serum | [69] |
| S100A9 | gastric juice, serum | [38,52] |
| SAA | serum | [56] |
| SAA1 | serum | [83] |
| sEGFR | plasma | [47] |
| SERPINA1 | serum | [87] |
| SERPINA4 | serum | [52] |
| SHBG | plasma | [49] |
| SIRT6 | serum | [57] |
| SLAMF8 | serum | [67] |
| SLC25A4 | urine | [93] |
| SNCG | gastric juice, serum | [59] |
| SORT1 | urine | [92] |
| SOX3 | serum | [64] |
| SRMS | serum | [78] |
| TERF1 | serum | [87] |
| TFF1 | urine | [94] |
| TGF-β1 | serum | [76] |
| THB1 | serum | [78] |
| TK1 | serum | [60,71] |
| TNFα | serum | [51,55,70] |
| TNFRSF19L | serum | [67] |
| TPI1 | saliva | [91] |
| TrxR | plasma | [46] |
| TSLP | plasma | [47] |
| VEGF | serum | [76,81] |
| VN | serum | [78] |
| VTN | urine | [92] |

^a)^ Protein name abbreviation; proteins in alphabetical order.
